# Ensemble-based multiclass lung cancer classification using hybrid CNN-SVD feature extraction and selection method

**DOI:** 10.1371/journal.pone.0318219

**Published:** 2025-03-19

**Authors:** Md. Sabbir Hossain, Niloy Basak, Md. Aslam Mollah, Md. Nahiduzzaman, Mominul Ahsan, Julfikar Haider

**Affiliations:** 1 Department of Electronics & Telecommunication Engineering, Rajshahi University of Engineering & Technology, Rajshahi, Bangladesh; 2 Department of Electrical & Computer Engineering, Rajshahi University of Engineering & Technology, Rajshahi, Bangladesh; 3 Department of Computer Science, University of York, York, United Kingdom; 4 Department of Engineering, Manchester Metropolitan University, Manchester, United Kingdom; Islamia University of Bahawalpur: The Islamia University of Bahawalpur Pakistan, PAKISTAN

## Abstract

Lung cancer (LC) is a leading cause of cancer-related fatalities worldwide, underscoring the urgency of early detection for improved patient outcomes. The main objective of this research is to harness the noble strategies of artificial intelligence for identifying and classifying lung cancers more precisely from CT scan images at the early stage. This study introduces a novel lung cancer detection method, which was mainly focused on Convolutional Neural Networks (CNN) and was later customized for binary and multiclass classification utilizing a publicly available dataset of chest CT scan images of lung cancer. The main contribution of this research lies in its use of a hybrid CNN-SVD (Singular Value Decomposition) method and the use of a robust voting ensemble approach, which results in superior accuracy and effectiveness for mitigating potential errors. By employing contrast-limited adaptive histogram equalization (CLAHE), contrast-enhanced images were generated with minimal noise and prominent distinctive features. Subsequently, a CNN-SVD-Ensemble model was implemented to extract important features and reduce dimensionality. The extracted features were then processed by a set of ML algorithms along with a voting ensemble approach. Additionally, Gradient-weighted Class Activation Mapping (Grad-CAM) was integrated as an explainable AI (XAI) technique for enhancing model transparency by highlighting key influencing regions in the CT scans, which improved interpretability and ensured reliable and trustworthy results for clinical applications. This research offered state-of-the-art results, which achieved remarkable performance metrics with an accuracy, AUC, precision, recall, F1 score, Cohen’s Kappa and Matthews Correlation Coefficient (MCC) of 99.49%, 99.73%, 100%, 99%, 99%, 99.15% and 99.16%, respectively, addressing the prior research gaps and setting a new benchmark in the field. Furthermore, in binary class classification, all the performance indicators attained a perfect score of 100%. The robustness of the suggested approach offered more reliable and impactful insights in the medical field, thus improving existing knowledge and setting the stage for future innovations.

## 1. Introduction

Cancer represents a collection of more than 100 distinct diseases characterized by abnormal cell proliferation, exceeding their typical confines, and the potential for metastasis. The primary cause of mortality in both males and females is lung cancer. It is a form of cancer that originates in the lung and involves the abnormal division of cells and impacts lung cells. According to statistics provided by the International Agency for Research on Cancer (IARC), approximately 19.3 million new cases of cancer and an estimated 10 million deaths related to cancer occurred in 2020 [1]. According to a report released by the American Cancer Society, the mortality rate for lung cancer is 26%, indicating a substantial fatality risk. Additionally, reports indicate that the five-year survival rate for individuals diagnosed with lung cancer is merely 18% [2]. From a pathological and treatment perspective, lung cancer (LC) can be classified into two major types: non-small cell lung carcinoma (NSCLC) and small cell lung carcinoma (SCLC). Approximately 80%-85% of these cases are NSCLC, whereas the remaining cases are SCLC [3]. NSCLC can be further classified into distinct histological types, with the primary types being lung adenocarcinoma (ADC) and lung squamous cell carcinoma (LUSC). Additional histological subtypes of NSCLC include lung adenosquamous carcinoma (ASC) and large-cell carcinoma [4]. Notably, ASC is a relatively uncommon subtype of NSCLC, representing approximately 0.3–5% of all NSCLC cases [5].

The essential customization of targeted treatment strategies relies on accurately classifying carcinoma types and evaluating their aggressiveness. In clinical settings, trained pathologists traditionally identify carcinoma by examining tissue slides stained with hematoxylin and eosin (H&E) under high-power microscopy. This process is both labor intensive and time intensive, with an experienced histopathologist typically dedicating approximately 15 minutes to half an hour to review a single whole-slide image (WSI). Furthermore, this approach often necessitates pathologists to scrutinize extensive areas of normal tissue before eventually identifying malignant regions. Additionally, many benign structures exhibit a similar appearance to cancerous regions, necessitating careful differentiation. Hence, there is a substantial demand for automated analysis techniques in the field of pathology. Such techniques have the potential to significantly alleviate workload, expedite the diagnostic process, and enable timely treatment interventions [6]. Nonetheless, the early detection of lung cancer using computed tomography (CT) has demonstrated the potential to significantly enhance patient survival rates [7].

The implementation of a Computer-Aided Diagnosis (CAD) system plays a pivotal role in delivering swift, precise, and effective disease diagnoses, thereby contributing significantly to enhanced patient care. The early detection of diseases has emerged as a crucial factor leading to a decline in mortality rates across a spectrum of cancers, including breast cancer, kidney stones, brain cancer, blood cancer, stomach cancer, and lung cancer. In response to this imperative, extensive research endeavors have been undertaken to advance and refine the diagnostic procedures for life-threatening diseases based on medical imagery, thus ushering in improved healthcare practices [8]. Several segmentation frameworks or models have been developed by researchers to aid radiologists in detecting cancerous lung tumors. These methods for lung cancer segmentation can be broadly classified into two main types: traditional techniques and deep learning (DL) techniques. Historically, conventional methodologies have predominantly revolved around intensity-based approaches, including but not limited to techniques such as region growing [9–11], adaptive thresholding [12–15], morphological procedures [16,17], active-contour models [18,19], and shape analysis [20,21].

Timely identification, precise prognosis, and diagnosis of lung cancer are crucial. Early detection and recognition of cancer play vital roles in increasing survival rates, improving recovery chances, and safeguarding human life. Diagnosing lung cancer from CT images is challenging because of the significant variance in lesion appearances and their resemblance to other lung diseases. To achieve high accuracy, machine learning models require large-scale databases that capture a diverse range of lesion patterns. Many existing studies use various machine learning and deep learning methods to classify lung cancers and achieve high accuracy. However, there is still scope for further improving accuracy, particularly for multiclass classification and visualizing the regions of interest that highlight the affected lesions by explainable AI. To resolve these problems and make it easier for lung cancer classification algorithms to be used in real-world situations, this paper proposes a Convolutional Neural Network-Singular Value Decomposition (CNN-SVD) framework with Ensemble learning. The novelty of the suggested model lies in the use of a hybrid CNN-SVD feature extraction and selection strategy to capture only important features, which enables more precise and effective lung cancer classification from CT scan images.

This study has the following contributions to be acknowledged.

(a) A hybrid ensemble structure, combining Convolutional Neural Networks (CNN) and Singular Value Decomposition (SVD), as CNN-SVD has been proposed for the extraction of prominent features from lung cancer CT scan images and subsequent classification.
(b) Classification performance enhancement was proposed through a boosting approach employing an ensemble technique that learns predictions from a diverse set of machine learning classifiers, including Support Vector Machine (SVM), k-Nearest Neighbors (KNN), Random Forest (RF), Gaussian Naive Bayes (GNB) and Gradient Boosting Machine (GBM).
(c) Gradient-weighted Class Activation Mapping (Grad-CAM) was also suggested to provide interpretability by visualizing the critical regions in CT scans that influence the model’s predictions, enhancing the system’s robustness and transparency.

The rest of this paper is organized as follows: Section 2 provides an overview of related work. The proposed method is detailed in Section 3. The experimental results are presented in Section 4, where the proposed method is evaluated using various metrics and compared with earlier models discussed in the literature. Finally, the conclusions are presented in Section 5.

## 2. Literature review

The manual detection of LC images has been fraught with challenges, primarily stemming from the shortage of expertise, particularly professional radiologists, and the high costs associated with these diagnostic procedures. This situation poses significant difficulties, especially for patients in less developed regions. In response to these challenges, there has been a concerted effort to develop automated processing techniques. The aim is to streamline access to precise and prompt diagnoses, particularly for early-stage patients. These automated approaches hold the promise of providing more accessible and efficient solutions for diagnosing and treating lung cancer, addressing critical healthcare needs.

In the past decade, CNNs have made remarkable strides in various domains within the medical imaging field. These domains include MRI [22,23] and CT [24]. Hussain *et al.* [25] suggested a hybrid approach based on texture, morphological, SIFT (Scale Invariant Feature Transform), GLCM (Gray Level Co-occurrence Matrix), entropy, Elliptic Fourier Descriptors (EFDs), RICA, and sparse filtering methods along with different machine learning classifiers, such as SVM, KNN, DT and Naïve Bayes. Their proposed model achieved the highest accuracy of 97.55% and an AUC of 99.76% with the RICA and SVM Gaussian kernels for breast cancer detection. This approach outperformed all the previous state-of-the-art methods for the same dataset, and the result behind their success was the ability to address the hidden features of images by utilizing the multimodal feature extraction strategy along with robust ML techniques. Hussain *et al.* [26] suggested an approach that uses the advantages of Deep Convolutional Neural Network (DCNN) along with GoogleNet and the AlexNet transfer learning model. Their approach lies with extracting some hand-crafted features first and then using them for different machine learning models. Then, they used CNN methods along with GoogleNet and AlexNet transfer learning models, which made their model more robust than the previous models. The suggested DCNN model has a considerable effect on the performance metrics, achieving 99.26% accuracy for both the GoogleNet and the AlexNet models. The reason behind their state-of-the-art performance with their suggested model was the dynamic feature engineering and extraction of the best features from the initial set of features. Naseer *et al.* [27] introduced the LUNA-16 dataset of CT scan images and utilized a U-Net-based segmentation and detection model to extract features and the Support Vector Machine (AlexNet-SVM) classification procedure to classify lung cancer from CT scan images, and the AlexNet-SVM model achieved 97.98% accuracy. Ozdemir *et al.* [28] introduced a 3D CNN model specifically crafted for identifying and diagnosing lung cancer through the analysis of low-dose CT scan images. Their model comprised two significant components, Computer-Aided Detection (CADe) and Computer-Aided Diagnosis (CADx) and their approach also incorporated model uncertainty, which contributed to increased robustness in real-world applications. The study achieved a commendable maximum AUC (Area Under the Curve) of 88.5%.

Khanna *et al.* [29] proposed a model based on the feature selection method along with three soft computing-based optimization algorithms during the training phase, which enhanced model effectiveness by incorporating discriminative features and eliminating irrelevant or redundant features. Their model achieved the state-of-the-art result with the highest accuracy of 97.96% using the Wisconsin Diagonostic Breast Cancer (WDBC) benchmark dataset, and the reason behind this commendable result is the use of feature selection techniques along with two optimization algorithmsnamed Teaching Learning-Based Optimization (TLBO), Elephant Herding Optimization (EHO), and a proposed hybrid algorithm of these two. They used a total of 11 features from the initial set of 32 features of the WDBC dataset using the TLBO algorithm, which allowed them with the best features to work with. All of their other performance metrics, such as precision (98.76%), F1 score (96.27%), specificity (98.73%) and sensitivity (98%), also outscored the previous state-of-the-art results on this dataset. Singh *et al.*[30] proposed a unique model utilizing the chest CT scan image dataset of COVID-19 patients. After pre-processing, their suggested model extracts 213 features but used only 75% of the initial features, which are more crucial for recognition via Chi-square set. These features were then forwarded to a cuckoo search optimization algorithm, a teaching–learning-based optimization algorithm, and a hybrid of these two for further optimization, and then the data were fed into four ML classifiers (SVM, KNN, RF, and XGBoost), which produced a notable accuracy of 95.99% and an AUC of 99.66%. Singh *et al.*[31] proposed a model that utilized the feature set by selecting a total of 36 most important features based on emperor penguin optimization, bacterial foraging optimization, and their hybrid model from the retinal fundus benchmark images and fed them into six transfer learning models. The hybrid model along with RF algorithm, achieved the highest accuracy of 95.41%. Despite using such fewer features, they were always able to obtain an accuracy greater than 80% in all the cases. Singh *et al.*[32] proposed a feature selection method by utilizing the hybrid approach of Eagle Strategy Optimization (ESO) and Gravitational Search Optimization (GSO) algorithm with the publicly available WDBC brast cancer dataset. Their work utilized soft computing methodologies along with machine learning strategies to create a hybrid model, which obtained an accuracy of 98.9578% and an AUC of 99.80%. The differences between benign and malignant breast cancer tumors were identified automatically through three feature selection algorithms and six classifiers by removing redundant and unnecessary features, which is crucial for improved performance. Although their model achieved significant success in addressing breast cancer identification and classification, it lacked in dealing with big data.

Shafi *et al*. [33] demonstrated a model based on deep learning approaches along with a SVM for the diagnosis of lung cancer. Their model detected changes in soft tissues first to recognize the presence of cancer and then classified the cancer nodules using SVM along with the maximum projection intensity (MIP) method and achieved an accuracy of 94%. Xie *et al.* [34] introduced a multi-view knowledge-based collaborative (MV-KBC) model which used deep neural networks for the classification of lung nodules from CT scan images. Their model learned to decompose a 3D nodule into nine fixed views, constructed a knowledge-based collaborative (KBC) submodel for each view, fine-tuned them using the ResNet-50 model and achieved an accuracy of 91.60% and AUC of 95.70%. Zhang and Kong [35] developed an efficient lung nodule detection model utilizing the Multi-Scene Deep Learning Framework (MSDLF) with the aid of a vesselness filter. This model increased the accuracy and decreased the false positive rate in the detection of four-stage lung cancer by effectively using a four-channel CNN architecture, and this paper shows that the MSDLF method has 98.7% efficiency. Chaunzwa *et al.* [36] suggested a radiomics approach for the prediction of non-small cell lung cancer (NSCLC) with the help of a CNN. They also demonstrated the use of transfer learning models such as VGG-16 and ResNet-50 and many machine learning algorithms such as KNN, SVM, and RF for classification. The VGG-16 model achieved an accuracy of 68.6%, with an AUC of 0.709. The best result, which was 76.5%, was extracted by applying both the kNN and SVM algorithms. Pang *et al.* [37] presented a deep learning model designed to identify various types of lung cancer from CT scan images. Their model used image augmentation to expand and balance the training data because the number of images was small and then used a Dense-Net network along with an adaptive boosting (AdaBoost) algorithm for classification, which achieved an accuracy of 89.85%, which was better than that of transfer learning models such as ResNet (accuracy = 80.25%), VGG-16 (accuracy = 76.42%) and AlexNet (accuracy = 63.98%). Sultan et al. [38] proposed a Multiscale Dilated Features Up-sampling Network (MDFU-Net) for brain tumor segmentation from heterogeneous and homogeneous brain data. The model achieved a Dice Similarity Coefficient (DC) of 62.66% and sensitivity of 51.98% for heterogeneous data, while for homogeneous data, it achieved a DC of 83.96% and sensitivity of 68.05%. Despite its success in handling heterogeneous brain tumor data, the model’s sensitivity for heterogeneous data remains low, highlighting limitations in diagnostic performance. Akram et al. [39] developed a segmentation model for crops and weeds using a modified U-Net, CED-Net, and data augmentation. Trained and tested on the BoniRob and CWFID datasets, the model achieved a mean IoU of 62% and an F1-score of 75.2% when trained on BoniRob and tested on CWFID, and 63.7% IoU with 74.3% F1-score when the datasets were swapped. While the model demonstrated robustness in heterogeneous environments, segmentation of small crops, weeds with similar colors, and thin regions remains challenging. Usman et al. [40] introduced a Dilated Multilevel Fused Network (DMLF-Net) for virus classification across three datasets: Matuszewski, Kylberg, and DIBaS. The model achieved 89.89% accuracy on the Matuszewski dataset, 80.70% on Kylberg, and 95.93% on DIBaS. Using a classification-driven retrieval framework with multilevel feature fusion, the model effectively classified 22 virus classes with minimal parameters (25.5 M). However, challenges persist with problematic samples in specific virus classes and the computational efficiency of the model.

Wang *et al.* [6] introduced an effective weakly supervised deep learning approach for classifying lung cancer using whole slide images. Their work was based on the utilization of the advantages of a fully convolutional network (FCN). The FCN is a fine tool for extracting deep features with high efficiency from CT images, and these features were fed into a RF classifier after performing feature aggregation, which achieved an accuracy of 97.3% and an AUC of 85.6%. Two multiple-resolution residually connected networks (MRRNs) were created by Jiang *et al*. [41] to segment lung nodules from CT images. The first one is the incremental MRRN, and the second one is the dense MRRN; both are multiscale CNN models and are used simultaneously for combining features from CT images. The suggested model was trained on 377 CT images from the TCIA dataset, validation was performed on the MSKCC dataset, and the model was tested on the LIDC dataset. This procedure is highly accepted and more robust than training, validation and testing on the same dataset. The segmentation accuracy was highest with the incremental-MRRN and Dice Similarity Coefficient (DSC) metric, which was 74% for the TCIA, 75% for the MSKCC and 68% for the LIDC. Li *et al.* [42] first extracted multidimensional features from histopathological images via a deep learning model and then used the Relief (Relevant Features) algorithm to select the best features. Finally, they applied the SVM algorithm for the classification of lung cancer subtypes. The Relief-SVM model achieved higher accuracy (83.91%) than the other mainstream classification models did.

Li *et al.* [43] introduced a generative-discriminative framework that includes two non-linear generative models to improve the generalizability of deep learning models, which were synchronized to assess lung cancer risk. The lung cancer risk prediction model achieved an AUC of 69.24% and a sensitivity of 76.54%. Kaviarasi *et al.* [44] suggested a prediction model for improving patient survival on the basis of a Gaussian classifier. This model showed that the Gaussian k-Base Naïve Bayes classifier has the better advancements and higher accuracy than existing models for the prediction of lung cancer. The results revealed that the Linear Regression and Naïve Bayes classifier had an AUC of 62.2% and 59.7%, respectively, whereas the Gaussian k-Base Naïve Bayes classifier had an AUC of 88.1%. Demiroğlu *et al*. [45] introduced a combined model of the DarknNet-53 and DenseNet-201 architectures for the assessment of multiclass classification of lung cancer using the Chest CT-Scan image dataset. The model uses Fine k-NN classifier and achieved 98.86% accuracy. Nahiduzzaman *et al*.[46] proposed a framework for lung cancer classification using a lightweight parallel depthwise separable CNN (LPDCNN) and ridge regression extreme learning machine (Ridge-ELM), which achieved an accuracy of 99% on CT images with SHapley Additive exPlanations (SHAP).

Previous studies have made many significant contributions to the field of lung cancer classification but further scientific advancements are still needed to facilitate better performance. For example, the authors of previous studies [28,36,37] used only CNNs to extract features, but this study aims to extract features using CNN-SVD architecture, which is more reliable and can extract the best available features present in the data. The authors of [28] suggested the building of a strategy that automatically rejected the most uncertain decision, which this research aimed to achieve. The authors of [47–49] mostly used the advantages of the CNN architecture to detect the early stages of lung cancer, which is directly related to this study, as this research also utilizes the advantages of CNNs and targets to obtain better outcomes than previous methods. In study [46], the authors suggested a prediction model utilizing the advancements of the Gaussian k-Base Naïve Bayes classifier, which this study also utilizes along with a diverse set of classifiers such as SVM, KNN, GBM and RF. In study [45], the authors used the advantages of DarkNet-53 and DenseNet-201 along with the Fine k-NN classifier but did not use all the data of the dataset, which was addressed in this study.

Prior research has concentrated on employing individual machine learning models for lung cancer classification, which may not be robust enough to manage the complexity and diversity of the data. Furthermore, the use of ensemble-based methods for classifying lung cancer has not been investigated in prior research, although these methods may be more successful in reducing error and enhancing overall prediction performance.

## 3. Proposed method

### 3.1. General architecture

**Fig 1:**
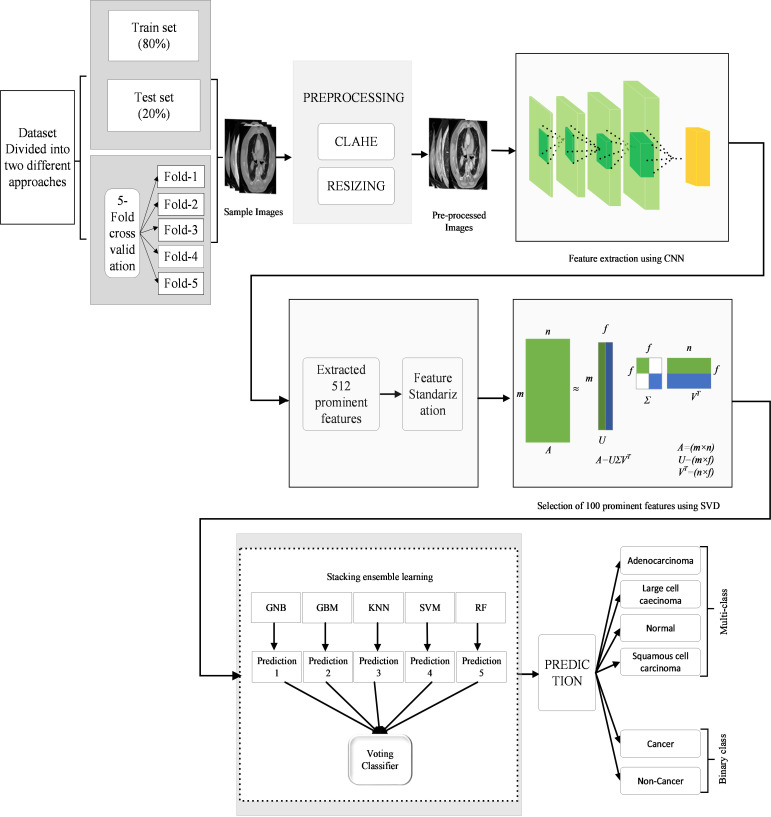
Structure of the proposed hybrid CNN-SVD-based ensemble model for lung cancer classification.

The general architecture of the suggested approach is depicted in Fig 1, which commences with a preprocessing step, where the CLAHE technique was applied to the input images while more recent techniques exist, such as Multiscale Retinex and Deep Learning-based enhancement, CLAHE offers a computationally efficient and robust solution, ensuring consistent image enhancement across diverse image conditions. This preprocessing step normalizes the images, effectively mitigating the impact of varying illumination conditions and intraclass variations, thereby improving the quality of the input images and enhancing feature extraction for downstream classification. The preprocessed images were then directed into a hybrid CNN-SVD model, which was specifically designed to extract and select discriminative features. This approach directly processes raw CT images without the need for segmentation, as the CNN-SVD framework is designed to learn and extract the most relevant features automatically. Once the features were extracted, they were subjected to further preprocessing before employing a set of well-established machine learning algorithms encompassing GNB, KNN, GBM, SVM, and RF. To increase the predictive power and robustness of the suggested model, a voting ensemble-based approach was introduced. This approach capitalizes on the collective wisdom of individual algorithms by aggregating their decisions through a majority voting scheme. By doing so, potential errors in any particular algorithm were effectively mitigated, thereby increasing the overall performance of the system. Overall, the proposed model uses a hybrid CNN-SVD feature extraction and selection method and a voting ensemble strategy that integrates the best features of several machine learning models to increase the accuracy and robustness of the classification findings.

### 3.2. Dataset and experimental set-up

The Chest CT-Scan image dataset, published in Kaggle [50] for a competition, serves to aid researchers without any associated cost. The dataset can be accessed directly via the following link: https://www.kaggle.com/datasets/mohamedhanyyy/chest-ctscan-images. This widely recognized dataset is extensively utilized for the detection of lung cancer. The data pose challenges due to variations in images, such as camera differences, eye polarity (left/right), inversion or view disparities, and the presence of noise-like artifacts and exposure issues. The dataset encompasses four classes: adenocarcinoma, large cell carcinoma, squamous cell carcinoma, and normal. The image data distributions for training and testing sets are presented in Table 1. The proposed ensemble method was subjected to two distinct training and testing approaches. In the first approach, a total of 988 CT scan images were employed, with 20% of these images allocated for testing and 80% allocated for training in both binary and multi-class classification scenarios. Subsequently, the performance of the proposed model was assessed using a 5-fold cross-validation procedure.

**Table 1:** Dataset distribution.

Classification type	Class Type	Training	Testing
Multiclass	Adenocarcinoma	264	68
	Large Cell Carcinoma	150	37
	Normal	162	41
	Squamous Cell Carcinoma	214	52
	**Total**	**790**	**198**
Binary	Cancer	628	157
	Non-cancer	162	41
	**Total**	**790**	**198**

To ensure transparency and reproducibility, the code of the proposed ensemble method is publicly available on GitHub repository: https://github.com/sabbir394/lung-cancer-classification. This enables other researchers to validate the findings and perform fair comparisons.

### 3.3. Pre-processing

#### 3.3.1. Enhancement of image contrast.

**Fig 2:**
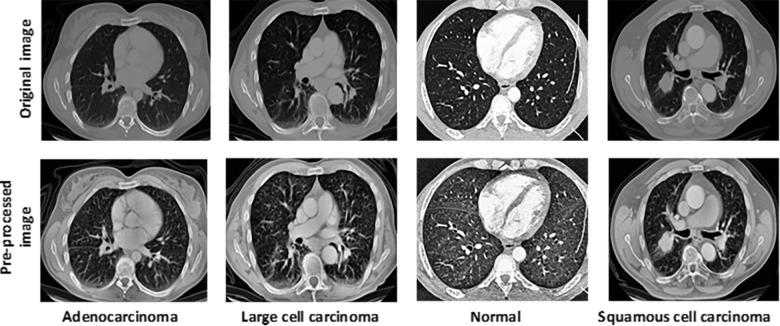
Lung cancer CT images before and after pre-processing.

To increase model accuracy, this study used Histogram Equalisation (HE) to increase visual contrast. Contrast, characterized by variations in color or brightness between objects within a given perspective, plays a vital role in distinguishing objects from their surroundings. This enhancement technique is particularly relevant to scientific image domains such as X-ray, satellite, and thermal imaging [51]. Given the utilization of CT scan images in this study, image contrast enhancement is of paramount importance. Specifically, the CLAHE technique, an extension of Adaptive Histogram Equalization (AHE), was employed. AHE breaks an image into smaller rectangular tiles and operates on each tile separately, whereas HE operates on the full image as a whole. This approach accentuates local contrast and edges but may exacerbate noise in regions with nearly uniform intensity [52]. CLAHE addresses this concern by limiting amplification through a clipping factor, thus achieving a balance between local contrast and edges. Despite conventional CT scan images being grayscale, the dataset utilized in this study, derived from the Kaggle lung cancer image dataset, is represented in three channels. Consequently, CLAHE can be effectively applied to both grayscale and color images [53]. In this investigation, contrast enhancement with CLAHE was performed, taking into account the ‘L’, ‘A’, and ‘B’ information channels. Therefore, the input data with three channels rather than one channel were employed. For this study, a clipping factor of 2.5 and a tile size of 8 × 8 were employed as the specific parameters for CLAHE. Fig 2 presents original and pre-processed lung CT scan images.

#### 3.3.2. Resizing.

The dataset comprises images of varying dimensions, which are resized to a standardized format for seamless integration into the CNN model. Following the application of CLAHE to the images, they are uniformly adjusted to dimensions of 224 × 224 pixels, optimizing their compatibility with the model.

### 3.4. Feature extraction and dimensionality reduction

The abundance of data, often characterized by high dimensionality, poses challenges in the development of efficient predictive models. Additionally, the computational expense associated with training such models can be substantial. Furthermore, certain features exhibit minimal variation across populations, providing limited informational value. Given the uneven distribution of information across dataset features, dimensionality reduction becomes imperative to enhance model efficiency and mitigate errors stemming from extraneous features. Notable techniques for achieving dimensionality reduction encompass Principal Component Analysis (PCA), Linear Discriminant Analysis (LDA), and Singular Value Decomposition (SVD). In this study, a combination of two feature engineering (FE) techniques was employed. Initially, a CNN model is utilized to condense the original 224 × 224 features into a reduced set of 512 features. SVD is subsequently applied to further reduce the dimensionality from 512 features to 100.

#### 3.4.1. Feature extraction using CNN.

Feature extraction constitutes a pivotal aspect of the classification problem, as the efficacy of a model hinges on the adeptness with which salient features are extracted from CT-Scan images. The extraction of discriminative features that effectively differentiate between classes is imperative to enhance the model’s classification performance. Feature extraction is a transformative technique that facilitates the conversion of high-dimensional data into a lower-dimensional format characterized by non-redundancy and informativeness [54]. This process not only enables streamlined data manipulation but also contributes to enhanced data management practices. To address the inherent complexity of feature extraction from CT-Scan images, a novel deep Convolutional Neural Network (CNN) was developed. This specialized network was designed to extract 512 key features, which were specifically tailored for lung cancer classification, given the intricate nature of the features present in these images. The CNN feature extractor model is characterized in Table 2.

**Table 2:** Parameter attributes of the CNN feature extractor model.

Name of Parameter	Attribute
Max-pooling Filter Size	2 × 2
Activation Function	ReLu
Dropout	0.5
Loss Function	categorical cross-entropy
Optimizer	Adam
Learning Rate	0.001
Batch Size	32
Epochs	100

**Fig 3:**
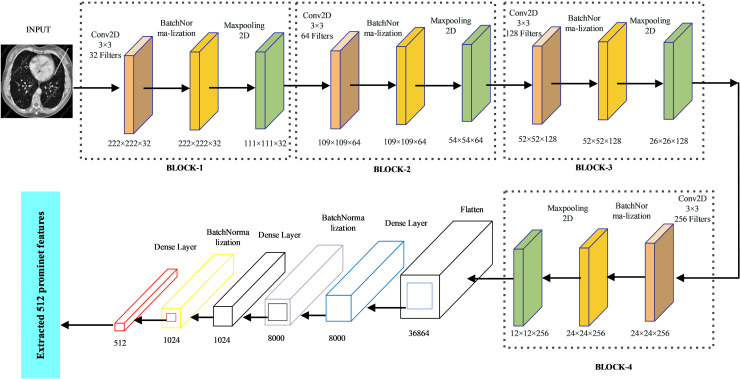
CNN structure for feature extraction.

Fig 3 illustrates the architecture of our proposed CNN model. The model comprises a total of four convolutional layers (CLs), with each CL followed by batch normalization and a max pooling layer. The integration of batch normalization is instrumental, as it expedites training and enhances model performance by re-centering and re-scaling the input data at each layer [55]. A max pooling layer with 2 × 2 filters was employed, facilitating the selection of the highest-value neuron within each cluster at the convolutional layers. This approach effectively extracts the most salient image components [56]. The ‘VALID’ padding strategy is employed, discarding border elements. To address the vanishing gradient problem, we employ Rectified Linear Unit (ReLU) as the activation function [56]. To mitigate overfitting, dropout regularization was incorporated to intermittently exclude random nodes within each layer during training, thereby substantially accelerating the training process. For optimization, Adam optimizer, which is renowned for its accuracy and suitability for CNNs, was employed particularly in large-scale data scenarios [57]. Finally, a final dense layer is employed to extract 512 discriminative features from each image. A summary of the deep CNN model is provided in Table 3.

**Table 3:** Overview of the proposed simple CNN for extracting features from CT scan images.

Layer (Type)	Output Shape	Parameters
conv2d_input (Input Layer)	(None, 224, 224, 3)	0
conv2d (Conv2D)	(None, 222, 222, 32)	896
batch_normalization	(None, 222, 222, 32)	128
activation (Activation)	(None, 222, 222, 32)	0
max_pooling2d (MaxPooling2D)	(None, 111, 111, 32)	0
conv2d_1 (Conv2D)	(None, 109, 109, 64)	18496
batch_normalization_1	(None, 109, 109, 64)	256
activation_1 (Activation)	(None, 109, 109, 64)	0
max_pooling2d_1 (MaxPooling2D)	(None, 54, 54, 64)	0
conv2d_2 (Conv2D)	(None, 52, 52, 128)	73856
batch_normalization_2	(None, 52, 52, 128)	512
activation_2 (Activation)	(None, 52, 52, 128)	0
max_pooling2d_2 (MaxPooling2D)	(None, 26, 26, 128)	0
conv2d_3 (Conv2D)	(None, 24, 24, 256)	295168
batch_normalization_3	(None, 24, 24, 256)	1024
activation_3 (Activation)	(None, 24, 24, 256)	0
max_pooling2d_3 (MaxPooling2D)	(None, 12, 12, 256)	0
flatten (Flatten)	(None, 36864)	0
dense (Dense)	(None, 8000)	294920000
batch_normalization_4	(None, 8000)	32000
activation_4 (Activation)	(None, 8000)	0
dropout (Dropout)	(None, 8000)	0
dense_1 (Dense)	(None, 1024)	8193024
batch_normalization_5	(None, 1024)	4096
activation_5 (Activation)	(None, 1024)	0
dropout_1 (Dropout)	(None, 1024)	0
dense_2 (Dense)	(None, 512)	524800

#### 3.4.2. Feature standardization and dimensionality reduction.

1) After extracting 512 features using the CNN, feature standardization was applied to ensure that each feature had a mean of zero and unit variance. This process is critical, as it standardizes the range of the features, preventing any single feature from dominating the analysis. Standardization was performed using the following equation:


$$\overset{´}{x}=\frac{x-\bar{x}}{\sigma }$$ (1)

where,

x = Original feature vector

$\bar{x}$= Mean of that feature vector

𝜎 = The standard deviation

Standardization is essential, as it influences the covariance matrix, which is a key component in the subsequent dimensionality reduction process.

Following standardization, SVD was applied to reduce the dimensionality of the feature space. SVD computes the covariance matrix 𝐴 = 𝑍∙𝑍𝑇, where 𝑍 is the standardized feature matrix. The eigenvalues and corresponding eigenvectors were then derived from this matrix. The top 100 principal components were selected based on the highest eigenvalues, thereby retaining the most significant variance within the data. The original 512-dimensional feature space was projected onto these eigenvectors, effectively reducing the dimensionality to 100 while preserving the most informative features for classification.

2) Singular Value Decomposition (SVD) is a data-driven technique based on the fundamental principle of the Fast Fourier Transform (FFT). In essence, Singular Value Decomposition (SVD) offers insights into the minimal dimensionality necessary for representing a matrix or linear transformation. In numerous instances, multidimensional data can be efficiently represented using fewer dimensions owing to inherent data redundancies. When a collection of n-dimensional vectors resides within a k-dimensional subspace (where k < n), each n-vector essentially possesses only k degrees of freedom, thereby enabling its unique characterization by k numerical values. This inherent characteristic aligns with the natural correlations observed in real-world data, rendering SVD a valuable candidate for feature reduction.

The SVD of a linear transformation *A*: $R^{n}$ → $R^{m}$is


$$A=U\Sigma V^{T}$$ (2)

In the SVD, the matrix *A* is factored into three constituent components: *U, Σ*, and $V^{T}$. Here, *U*: $R^{n}$ → $R^{m}$ represents the left singular vectors of *A* and is an orthogonal matrix ($U^{T}U=I$). The diagonal matrix $A,\Sigma :R^{n}\to R^{n}$comprises singular values associated with *A*. Additionally, $V^{T}:R^{n}\to R^{n}$. represents the right singular vectors of *A* and is also orthogonal. Every linear transformation in the SVD is expanded in the following manner, assuming *m <  n*:


$$\begin{array}{l} \left(\begin{array}{c} a_{11}\\ \vdots \\ a_{m1} \end{array}\begin{array}{ccc} a_{12} & \cdots & a_{1n}\\ \vdots & \ddots & \vdots \\ a_{m2} & \cdots & a_{mn} \end{array}\right)=\left(\begin{array}{ccc} u_{11} & \cdots & u_{1m}\\ \vdots & \ddots & \vdots \\ u_{m1} & \cdots & u_{mm} \end{array}\right)\left(\begin{array}{c} \sigma_{11}\\ \vdots \\ \sigma_{m1} \end{array}\begin{array}{ccc} \sigma_{12} & \cdots & \sigma_{1n}\\ \vdots & \ddots & \vdots \\ \sigma_{m2} & \cdots & \sigma_{mn} \end{array}\right)\\ \times \left(\begin{array}{c} v_{11}\\ \vdots \\ v_{m1} \end{array}\begin{array}{ccc} v_{12} & \cdots & v_{1n}\\ \vdots & \ddots & \vdots \\ v_{m2} & \cdots & v_{mn} \end{array}\right) \end{array}$$ (3)

Since the entries $\sigma_{ij}$ when *j >  m* are all zero, the product $\Sigma V^{T}$ will produce entries of zero for rows *m + *1 through *n*. The SVD may be rewritten as


$$\left(\begin{array}{c} a_{11}\\ \vdots \\ a_{m1} \end{array}\begin{array}{ccc} a_{12} & \cdots & a_{1n}\\ \vdots & \ddots & \vdots \\ a_{m2} & \cdots & a_{mn} \end{array}\right)=\left(\begin{array}{ccc} u_{11} & \cdots & u_{1m}\\ \vdots & \ddots & \vdots \\ u_{m1} & \cdots & u_{mm} \end{array}\right)\left(\begin{array}{ccc} \sigma_{1} & 0 & 0\\ 0 & \ddots & 0\\ 0 & 0 & \sigma_{m} \end{array}\right)$$

$$\times \left(\begin{array}{c} v_{11}\\ \vdots \\ v_{m1} \end{array}\begin{array}{ccc} v_{12} & \cdots & v_{1n}\\ \vdots & \ddots & \vdots \\ v_{m2} & \cdots & v_{mn} \end{array}\right)$$ (4)

This demonstrates that a column $A_{i}$ of *A*, which is an *m*-dimensional vector, can be expressed as a linear combination of the m basis vectors in *U* ($U_{1}$*,* $U_{2}$,..., $U_{m}$), utilizing the singular values in *Σ* ($\sigma_{1}$*,* $\sigma_{2}$,..., $\sigma_{m}$), along with the ith column $V_{i}^{T}$ in $V^{T}$. The diagonal elements of *Σ* are non-negative and can be arranged in descending order such that $\sigma_{1}$ ≥  $\sigma_{2}$ ≥  · · · ≥  $\sigma_{m}$. In cases where certain entries on the diagonal of *Σ* are zero, there exists a value *k* such that $\sigma_{1}$ ≥  $\sigma_{2}$ ≥  · · · ≥  $\sigma_{k}$,>  $\sigma_{k+1}$, =  · · · =  $\sigma_{m}$ = 0. Applying the aforementioned logic, which facilitates a reduction in the number of rows in $V^{T}$ from *n* to *m*, we can similarly reduce the number of columns in *U* to *k*, adjust the number of rows and columns of *Σ* to *k*, and further decrease the number of rows in $V^{T}$ to k., yielding


$$\left(\begin{array}{c} a_{11}\\ \vdots \\ a_{m1} \end{array}\begin{array}{ccc} a_{12} & \cdots & a_{1n}\\ \vdots & \ddots & \vdots \\ a_{m2} & \cdots & a_{mn} \end{array}\right)=\left(\begin{array}{ccc} u_{11} & \cdots & u_{1k}\\ \vdots & \ddots & \vdots \\ u_{m1} & \cdots & u_{mk} \end{array}\right)\left(\begin{array}{ccc} \sigma_{1} & 0 & 0\\ 0 & \ddots & 0\\ 0 & 0 & \sigma_{k} \end{array}\right)$$

$$\times \left(\begin{array}{c} v_{11}\\ \vdots \\ v_{k1} \end{array}\begin{array}{ccc} v_{12} & \cdots & v_{1n}\\ \vdots & \ddots & \vdots \\ v_{k2} & \cdots & v_{kn} \end{array}\right)$$ (5)

An operation, such as classification, typically executed on the entire *m ×  n* matrix *A,* can now be equally accomplished on the entire *k × n* matrix $\Sigma V^{T}$ where *k < m*, leading to a decrease in the number of bands present in each vector [58].

#### 3.4.3. Gaussian naïve Bayes (GNB).

GNB is a probabilistic classification algorithm that incorporates the principles of Naïve Bayes and the Gaussian Normal Distribution. Naïve Bayes belongs to a class of supervised machine learning classification algorithms rooted in Bayes’ Theorem. In essence, it encapsulates the relationship between prior probabilities and posterior probabilities, facilitating effective classification. The “*Bayes Theorem”* is used to calculate the conditional probability and is defined as


$$P\left(AB\right)=\frac{P\left(A\cap B\right)}{P\left(B\right)}=\frac{P\left(A\right)\cdot P\left(BA\right)}{P\left(B\right)}$$ (6)

The variable *A* is the class variable, and the variable *B* represents the features. where *B = (*$b_{1}$, $b_{1}$,... $b_{n}$*)*

The Gaussian Naïve Bayes (GNB) is defined as


$$P\left(x_{i}|y\right)=\frac{1}{\sqrt{2\pi \sigma_{y}^{2}}}\exp\left(-\frac{{\left(x_{i}-\mu_{y}\right)}^{2}}{2\sigma_{y}^{2}}\right)$$ (7)

where *x*_*i*_ = Variable, *y* = class, $\mu_{y}$ = mean, and $\sigma_{y}$= standard deviation.

#### 3.4.4. k-nearest neighbors (kNN).

KNN algorithm is a fundamental machine learning technique used for both classification and regression tasks. It operates by measuring the distance between a data point of interest and its neighboring data points in a feature space. By selecting the ‘K’ nearest neighbors based on these distances, the algorithm assigns a class label (for classification) or predicts a value (for regression) for the data point. K-NN is intuitive and does not assume a specific data distribution. It is widely applicable but sensitive to the choice of ‘K’ and can be computationally intensive for large datasets. Nonetheless, it is a valuable tool for various machine learning applications.

#### 3.4.5. Gradient boosting machine (GBM).

GBM is the most powerful boosting algorithm present in machine learning for classification and regression problems. GBM works with the help of building some weak learners and fixing the errors of the prior learners, and ultimately, it combines the predictions from weak learners to generate final predictions. The GBM approximates with two steps. First, it fits $f(x;\tau_{m})$ as follows:


$$\tau_{m}=\text{arg}\underset{\tau }{\text{min}}\overset{n}{\underset{i=1}{\sum }}{\left(g_{im}-f\left(x_{i};\tau \right)\right)}^{2}$$ (8)

where


$$g_{im}=-{\left[\frac{\partial \text{Φ}\left(y_{i},F\left(x_{i}\right)\right)}{\partial F\left(x_{i}\right)}\right]}_{F\left(x\right)=F_{m-1}\left(x\right)}$$ (9)

Second, it learns *ρ* by


$$\rho_{m}=\text{arg}\underset{\rho }{\text{min}}\overset{n}{\underset{i=1}{\sum }}\text{Φ}\left(y_{i},F_{m-1}\left(x_{i}\right)+\rho f\left(x_{i};\tau_{m}\right)\right)$$ (10)

Then, it updates $F_{m}\left(x\right)=F_{m-1}\left(x\right)+\rho_{m}f\left(x;\tau_{m}\right)$. In practice, however, shrinkage is often introduced to control overfitting, and the update becomes $F_{m}\left(x\right)=F_{m-1}\left(x\right)+v\rho_{m}f\left(x;\tau_{m}\right)$, where 0<v≤1.

#### 3.4.6. Support Vector Machine (SVM).

SVM is a supervised machine learning algorithm tailored to handle both classification and regression tasks. Its classification process involves identifying a hyperplane that effectively separates different classes. SVM achieves this by maximizing the margin, which is the space between the hyperplane and the nearest data points of each class. To address non-linearity in data, the SVM employs the kernel trick. This technique transforms the low-dimensional input space into a higher-dimensional space, making previously inseparable problems separable. This feature is particularly advantageous for addressing non-linearly separable problems. In this study, sigmoid kernel function was utilized to facilitate the effectiveness of the SVM in handling such scenarios.

#### 3.4.7. Random forest (RF).

Random Forest (RF) is a supervised learning algorithm known for its ensemble technique, wherein it constructs a ‘forest’ comprising multiple decision trees. These decision trees are typically trained using the ‘bagging’ method, which is based on the principle that amalgamating diverse learning models enhances the final predictive performance. RF mitigates the overfitting issue commonly associated with individual decision trees by creating multiple decision trees during training [59]. The final prediction from RF is determined through methods such as class mode or averaging the predictions made by each individual tree. This technique effectively addresses the overfitting problem encountered when training decision trees, ensuring robust and reliable outcomes.

Feature importance score for feature *X*:


$$FI\left(X\right)=\overset{T}{\underset{t=1}{\sum }}\left(\frac{\left|D_{t}\right|}{\left|D\right|}\right)\text{Δ}I\left(X,D_{t}\right)$$ (11)

where

$FI\left(X\right)$is the feature importance score for feature *X* and where T is the number of trees in the Random Forest.$D_{t}$ is the dataset used by tree *t*.$\text{ Δ}I\left(X,D_{t}\right)$ is the reduction in impurity due to splitting on feature *X* in tree *t*.

#### 3.4.8. Ensemble learning.

The ensemble model is a strategic amalgamation of base models designed to create a robust and highly effective model. It leverages a diverse array of learning algorithms to address complex classification and regression challenges that individual models may struggle with. Ensemble learning [60] harnesses the strengths of multiple models, resulting in superior classification accuracy compared with single models. It is possible for individual models to overfit the training set, capturing noise or particular patterns that are poorly generalized to fresh, unseen data. Since various models tend to overfit in different ways, the ensemble technique aids in mitigating overfitting and improving generalizability.

In this study, soft voting ensemble learning was employed. Initially, the base models, including GNB, KNN, GBM, SVM and RF utilizing were trained with the training dataset. Subsequently, the performance of these models was evaluated using a separate test dataset, resulting in individual predictions by each model. These individual model predictions serve as supplementary inputs to our ensemble learning approach, which functions as a unified model trained to produce the final prediction.

## 4. Experimental results and analysis

The proposed hybrid CNN-SVD-based Ensemble network for lung cancer classification was evaluated on the Chest CT-Scan image dataset published in Kaggle. This section presents details of the dataset, performance measurement metrics, experimental set-up, and classification results.

### 4.1. Performance metrics applied


$$Accuracy=\frac{TP+TN}{TP+TN+FP+FN}$$ (12)

The performance of the proposed method is evaluated in relation to other approaches through metrics such as precision, accuracy, f1-score, recall, Cohen’s kappa, Matthews correlation coefficient (MCC) and the area under the ROC curve. These metrics are computed mathematically in the following manner:


$$Recall=\frac{TP}{\left(TP+FN\right)}$$ (13)

$$F1-Score=\frac{2*\left(Precision*Recall\right)}{\left(Precision+Recall\right)}$$ (14)

$$Precision=\frac{TP}{\left(TP+FP\right)}$$ (15)

$$MCC=\frac{TP\times TN-FP\times FN}{\sqrt{\left(TP+FP\right)\left(TP+FN\right)\left(TN+FP\right)\left(TN+FN\right)}}$$ (16)

$$Kappa=\frac{p_{0}-p_{e}}{1-p_{e}}$$ (17)

$$p_{e}=\overset{C}{\underset{i=1}{\sum }}\left(\frac{\left(a_{i}-b_{i}\right)}{N^{2}}\right)$$ (18)

In this context, $p_{0}$ is the observed agreement between the two raters, $p_{e}$ is the expected agreement due to chance, calculated as the product of the marginals of each class, $a_{i}$ is the sum of the observations in row *i*, $b_{i}$ is the sum of the observations in column *i*, *N* is the total number of instances, true positive (TP) represents the count of images correctly classified as the positive class, true negative (TN) denotes the number of samples accurately classified as the negative class, false positive (FP) signifies instances originally labeled as negative but misclassified as positive, and false negative (FN) indicates instances belonging to the positive class but predicted as negative. The ROC curve is constructed with the true positive rate on the x-axis and the false positive rate on the y-axis. The AUC is computed as the area underneath this curve.

### 4.2. Results for multiclass lung cancer classification

**Fig 4:**
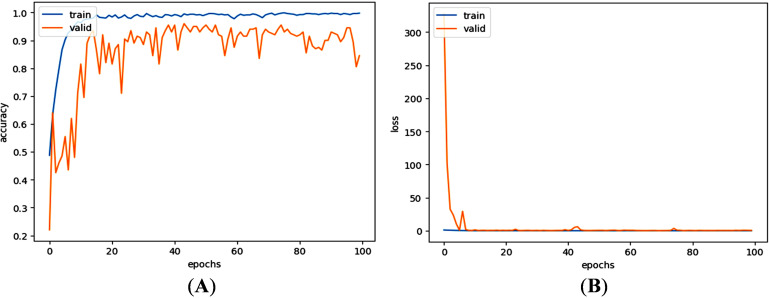
(A) Accuracy and (B) loss of training and validation sets of multiclass classification using CNN.

In Fig 4, the training and validation accuracy results after applying the CNN to the dataset for feature extraction are presented for the multiclass LC classification, achieving accuracies of 97.02% and 82.24%, respectively. During this dimensionality reduction process, the training and validation losses for CNN feature extraction on this dataset were measured as 0.0845 and 7.5670, respectively.

**Fig 5:**
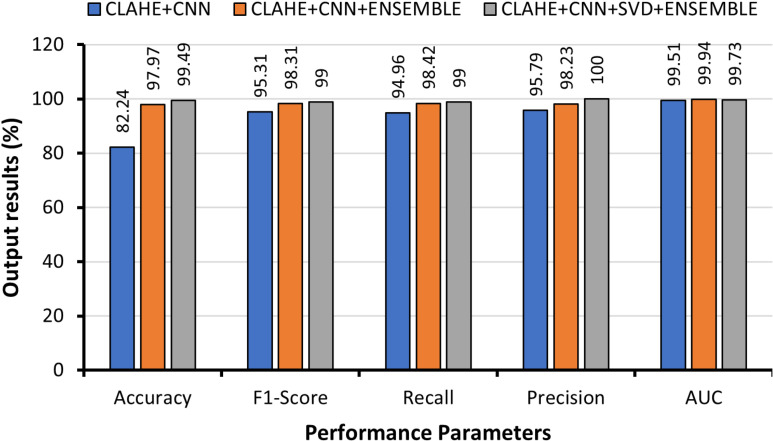
Graphical comparison of multiclass lung cancer classification with different approaches.

Fig 5 visually presents a comparative analysis of the performance metrics among CLAHE-CNN, CLAHE-CNN-ENSEMBLE, and CLAHE-CNN-SVD-ENSEMBLE for multiclass classification. This graphical representation underscores the significant performance improvement achieved by processing images with CLAHE and extracting features via the hybrid CNN-SVD approach. This not only significantly enhanced model performance but also effectively reduced the number of features, leading to reduced computational complexity and cost. Notably, Ensemble with hybrid CNN-SVD consistently achieved the highest scores across all the metrics.

**Fig 6:**
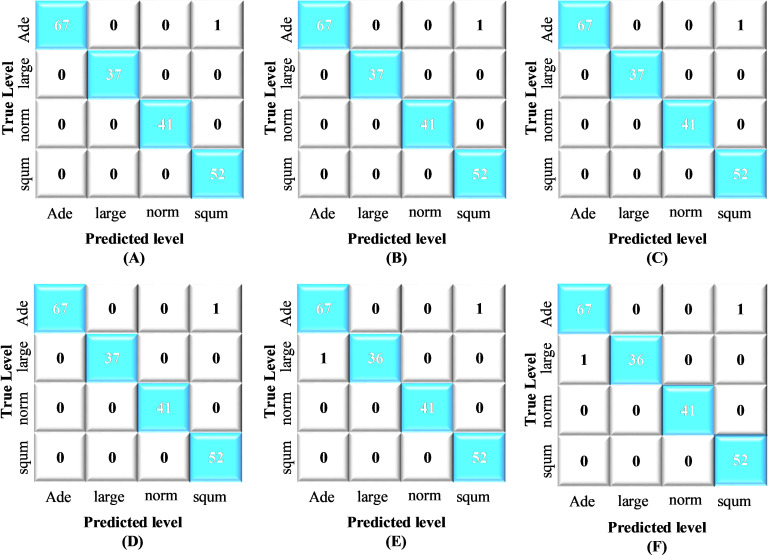
Normalized confusion matrix of the proposed method for multiclass classification: (A) GNB, (B) GBM, (C) KNN, (D) SVM, (E) RF, (F) Ensemble LEARNING.

**Fig 7:**
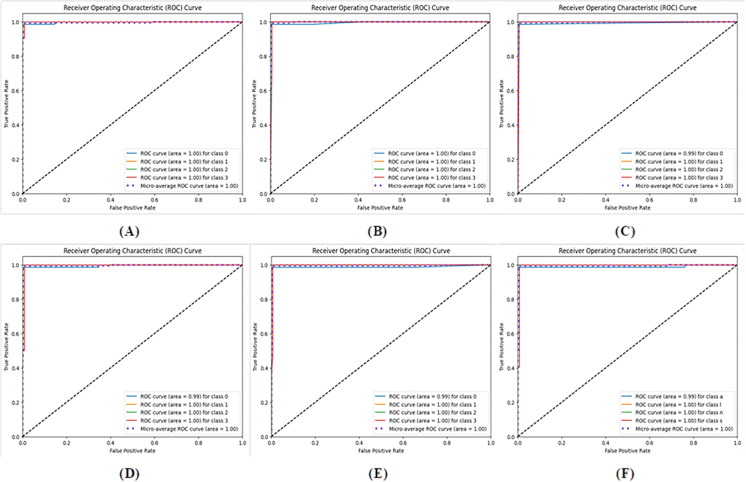
ROC curves of the proposed method for multiclass classification: (A) GNB, (B) GBM, (C) KNN, (D) SVM, (E) RF, and (F) Ensemble learning.

Fig 6 presents a normalized confusion matrix, offering deeper insight into the model’s accuracy in classifying various lung cancer classes. This confusion matrix was generated by applying the proposed method to the test dataset. The precision, recall, F1 score, and accuracy were subsequently calculated for the ensemble model on the basis of the confusion matrix. Fig 7 shows the area under the curve (AUC) for the hybrid CNN-SVD-based Ensemble network in the classification of four lung cancer stages. This metric serves as a crucial measure for assessing and optimizing the performance of machine learning models. Table 4 provides a comprehensive overview of the classification performance measures for all the models. Notably, the exceptional ability of the proposed method to accurately identify normal CT scan images (indicating the absence of lung cancer) with 100% accuracy is a highly desirable outcome in real-world applications. In this context, 512 features were extracted via a CNN, and an ensemble-based machine learning approach was subsequently applied to classify the four stages of lung cancer. The average accuracy scores for the GNB, GBM, KNN, SVM, RF, and Ensemble models were 99.49%, 99.49%, 99.49%, 99.49%, 98.98%, and 99.49%, respectively.

**Table 4:** Performance of the individual and proposed methods for multiclass classification of lung cancer.

Model Name	Class Type	Precision	Recall	F1-Score	AUC	Accuracy	Cohen’s Kappa	MCC
GNB	Adenocarcinoma	100%	99%	99%	–	–		
	Large cell carcinoma	100%	100%	100%	–	–		
	Normal	100%	100%	100%	–	–		
	Squamous cell carcinoma	98%	100%	99%	–	–		
	Average (micro)	100%	99%	99%	99.92%	99.49%	100%	100%
GBM	Adenocarcinoma	100%	99%	99%	–	–		
	Large cell carcinoma	100%	100%	100%	–	–		
	Normal	100%	100%	100%	–	–		
	Squamous cell carcinoma	98%	100%	99%	–	–		
	Average (micro)	100%	99%	99%	99.77%	99.49%	99.15%	99.16%
SVM	Adenocarcinoma	100%	100%	99%	–	–		
	Large cell carcinoma	100%	100%	100%	–	–		
	Normal	100%	100%	100%	–	–		
	Squamous cell carcinoma	98%	100%	99%	–	–		
	Average (micro)	100%	99%	99%	99.78%	99.49%	99.15%	99.16%
KNN	Adenocarcinoma	100%	99%	99%	–	–		
	Large cell carcinoma	100%	100%	100%	–	–		
	Normal	100%	100%	100%	–	–		
	Squamous cell carcinoma	98%	100%	99%	–	–		
	Average (micro)	100%	99%	99%	99.72%	99.49%	99.15%	99.16%
RF	Adenocarcinoma	99%	99%	99%	–	–		
	Large cell carcinoma	100%	97%	99%	–	–		
	Normal	100%	100%	100%	–	–		
	Squamous cell carcinoma	98%	100%	99%	–	–		
	Average(micro)	99%	99%	99%	99.59%	98.98%	99.15%	99.16%
Ensemble Learning	Adenocarcinoma	100%	99%	99%	–	–		
	Large cell carcinoma	100%	100%	100%	–	–		
	Normal	100%	100%	100%	–	–		
	Squamous cell carcinoma	98%	100%	99%	–	–		
	Average (micro)	**100%**	**99%**	**99%**	**99.73%**	**99.49%**	**99.15%**	**99.16%**

**Fig 8:**
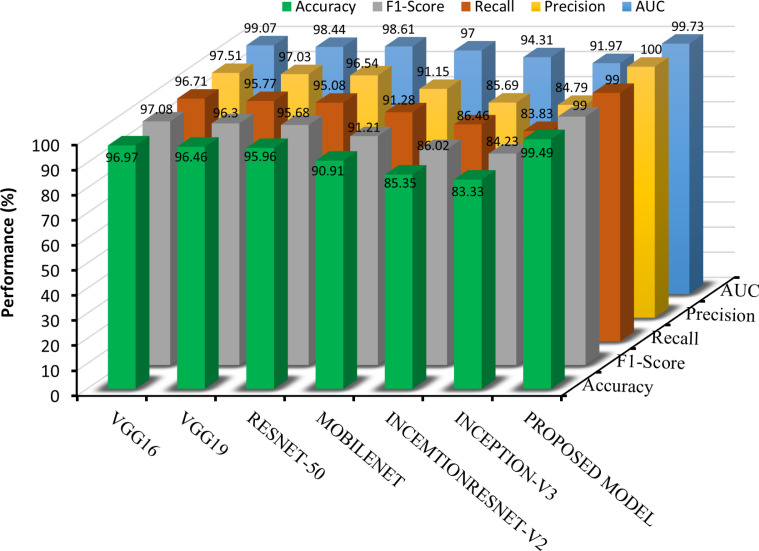
Performance comparison between the proposed method and various TL models with SVM.

To ensure a thorough comparison of the performance assessment for multi-class classification, well-known deep structures such as VGG-16, VGG-19, RESNET-50, MOBILENET, INCEPTION-V3, INCEPTIONRESNET-V2 and INCEPTION V3 models were also trained. To this end, in each model, the last fully connected layer was modified, and the models were trained for 100 epochs with the same setting. The transfer learning models were used to extract image features for further classification, and the features were used as input to the SVM classifier. The results of the comparison for the dataset are illustrated in Fig 8. Since SVM is well-known for handling high-dimensional data and non-linearly separable problems, image classification tasks are a good fit. SVM was used during experimentation because of its simplicity and strong theoretical foundation. Additionally, SVM is frequently used as a benchmark technique for comparison with other classifiers because of its reliable performance on a variety of classification problems. Compared with the best baseline model (VGG-16-SVM), the proposed method demonstrated superior performance. Specifically, a noteworthy improvement of 2.52% in the accuracy metric was observed. Furthermore, compared with other modified models, including VGG19, RESNET-50, MOBILENET, INCEPTIONRESNET-V2, and INCEPTION-V3, the proposed method achieved remarkable accuracy enhancements of 3.03%, 3.53%, 8.58%, 14.14%, and 16.16%, respectively.

### 4.3. Results for binary class lung cancer classification

**Fig 9:**
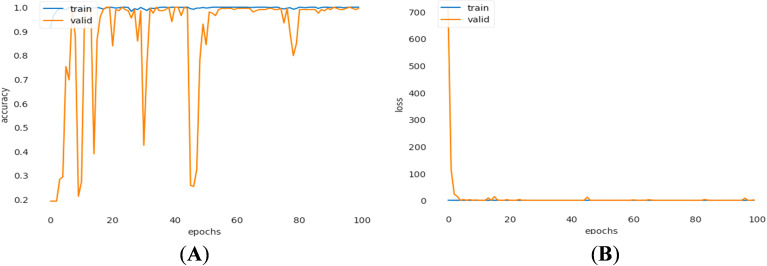
(a) Accuracy and (b) loss curves of training and validation during binary class classification for 512 features extracted by the CNN.

For binary lung cancer classification, a similar approach to multiclass classification has been employed. Fig 9 shows the training and validation accuracies, as well as the losses observed during the feature extraction phase for binary class classification. The fluctuations in Fig 9(a) could be due to the unnecessarily large number of initial features (512) extracted by the CNN and certain variability in the training process with a specific set of data, hyperparameter settings, and task complexity in classification. However, after reducing 512 CNN-extracted features to 100 significant ones by refining through SVD, the fluctuations could be removed, as evidenced by the high classification performance for the binary class. Remarkably, the training accuracy reached 99.61%, with a corresponding validation accuracy of 88.16%. The associated training and validation losses were recorded at 0.0155 and 8.6465, respectively.

**Fig 10:**
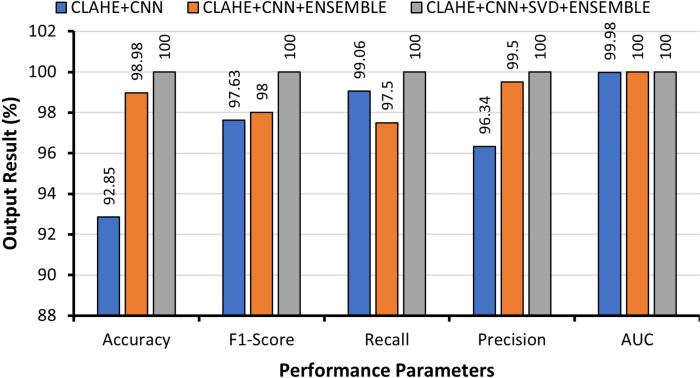
Graphical comparison of binary class lung cancer classification with different approaches.

Fig 10 provides a performance comparison of various evaluation metrics among CLAHE-CNN, CLAHE-CNN-ENSEMBLE, and CLAHE-CNN-SVD-ENSEMBLE for binary classification. It was evident that the use of hybrid CNN-SVD as a feature extractor significantly enhanced the performance of the Ensemble model.

**Fig 11:**
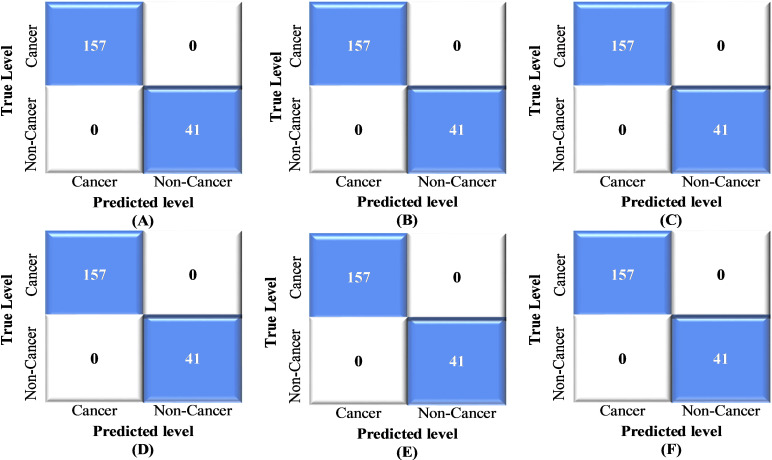
Normalized confusion matrix of the proposed method for multiclass classification: (A) GNB, (B) GBM, (C) KNN, (D) SVM, (E) RF, and (F) Ensemble learning.

**Fig 12:**
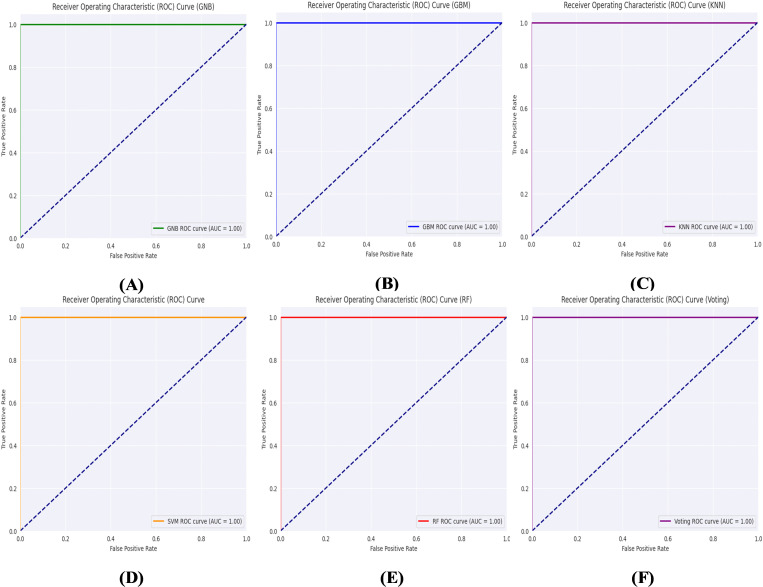
ROC curves of the proposed method for binary class classification: (A) GNB, (B) GBM, (C) KNN, (D) SVM, (E) RF, and (F) Ensemble learning.

Fig 11 presents a normalized confusion matrix (CM) to assess the accuracy of the binary classification of lung cancer. Fig 12 illustrates the Receiver Operating Characteristic (ROC) curves of the proposed method for binary class classification. Table 5 shows the outcomes of the proposed hybrid CNN-SVD-based ensemble model. The average accuracies of the GNB, GBM, KNN, SVM, RF, and Ensemble models reached 100% for each, demonstrating remarkable classification accuracy.

**Table 5:** Performance of the proposed method for binary classification of lung cancer.

Model Name	Class Type	Precision	Recall	F1-Score	AUC	Accuracy	Cohen’s Kappa	MCC
GNB	Cancer	100%	100%	100%	–	–		
	Non-Cancer	100%	100%	100%	–	–		
	Average	100%	100%	100%	100%	100%	100%	100%
GBM	Cancer	100%	100%	100%	–	–		
	Non-Cancer	100%	100%	100%	–	–		
	Average	100%	100%	100%	100%	100%	100%	100%
SVM	Cancer	100%	100%	100%	–	–		
	Non-Cancer	100%	100%	100%	–	–		
	Average	100%	100%	100%	100%	100%	100%	100%
KNN	Cancer	100%	100%	100%	–	–		
	Non-Cancer	100%	100%	100%	–	–		
	Average	100%	100%	100%	100%	100%	100%	100%
RF	Cancer	100%	100%	100%	–	–		
	Non-Cancer	100%	100%	100%	–	–		
	Average	100%	100%	100%	100%	100%	100%	100%
ENSEMBLE LEARNING	Cancer	100%	100%	100%	–	–		
	Non-Cancer	100%	100%	100%	–	–		
	Average	**100%**	**100%**	**100%**	**100%**	**100%**	**100%**	**100%**

### 4.4. K-fold validation of results

Table 6 outlines the results obtained from the 5-fold cross-validation process, which was carried out for both binary and multi-class classification tasks. The 5-fold cross-validation result showed poorer performance than the proposed method did. The reason behind this was that the model might become overfit or underfit after several iterations of training and testing on multiple data subsets. The proposed method, on the other hand, only included one training and one testing datasets. If the training set was not representative of the entire dataset, this might result in a less accurate prediction of the model’s performance. The particular setup associated with the cross-validation process or the selection of hyperparameters might have an impact on the suggested approach. For instance, the accuracy of the model might be impacted by the number of folds employed in the cross-validation procedure; in general, higher numbers of folds result in more accurate estimations of the model’s performance.

**Table 6:** Evaluation of performance across various settings.

Classification type	Setting	Accuracy	Precision	Recall	F1-Score
Multiclass	CLAHE-CNN-SVD-ENSEMBLE with (Proposed method: 80% training and 20% testing dataset)	99.49%	100%	99%	99%
	CLAHE-CNN-SVD-ENSEMBLE with 5-fold cross validation	98.48%	98.54%	98.49%	98.5%
Binary	CLAHE-CNN-SVD-ENSEMBLE with (Proposed method: 80% training and 20% testing dataset)	100%	100%	100%	100%
	CLAHE-CNN-SVD-ENSEMBLE with 5-fold cross validation	98.98%	99.37%	97.5%	98.40%

### 4.5. Explainability through Grad-CAM visualization

**Fig 13:**
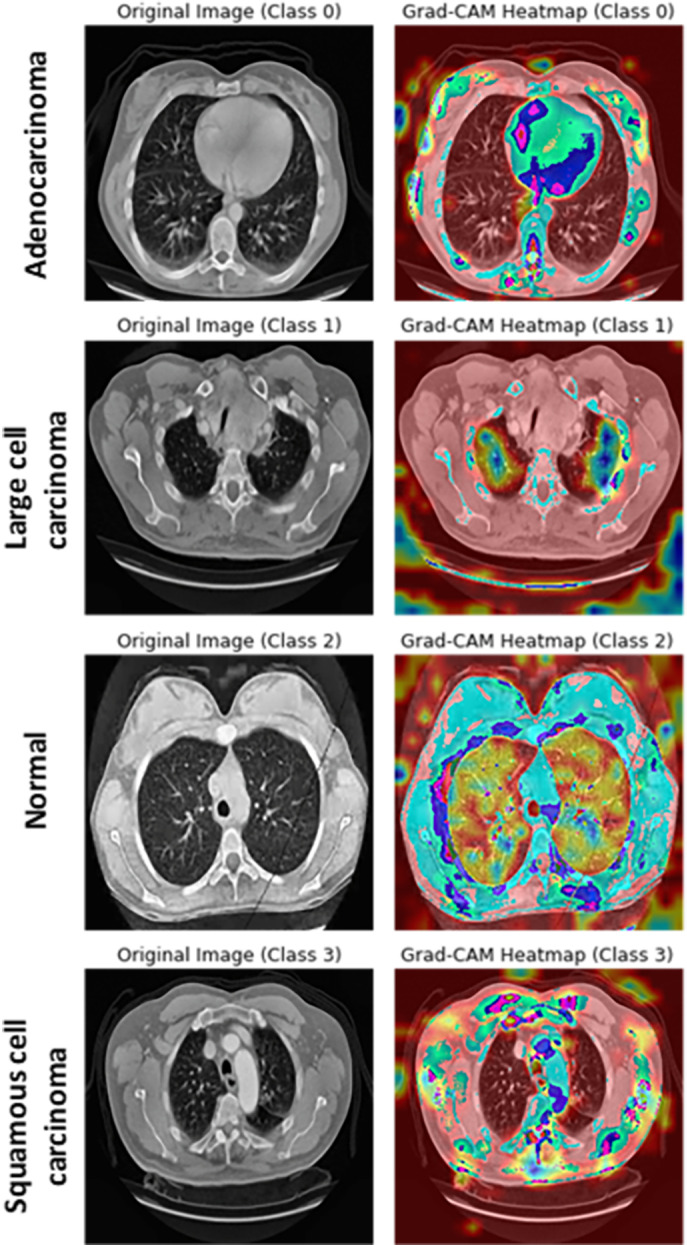
Visualization of color maps using Grad-CAM for different lung cancer classes.

To validate the robustness and interpretability of the proposed lung cancer classification model, Gradient-weighted Class Activation Mapping (Grad-CAM) was employed to visualize the critical regions that the CNN-based model focuses on during classification. As shown in Fig 13, the Grad-CAM heatmaps highlight the areas of the lung CT images that are most influential in the model’s decision-making process. For correctly classified cases, strong activation is consistently observed around the suspicious lesions, particularly in tumor areas, confirming that the model detected the discriminating features relevant to the lung cancer diagnosis.

### 4.6. Comparison with the literature

The proposed CNN-SVD ensemble model significantly outperforms prior SOTA models, as summarized in Table 7. In binary classification, Naseer *et al*. [27] obtained high precision, f1-scores, and accuracy, with values of 97.53%, 97.70%, and 97.98%, respectively, but the suggested framework exceeded their findings, achieving a perfect accuracy of 100%. Pang *et al*. [37] and Han *et al*. [3] used different datasets for 3-class and 2-class classifications, but in both cases, accuracies less than 90% were achieved. These two studies, along with the study by Wang *et al*. [6], used datasets different from the dataset used in this study.

**Table 7:** Result comparison with other existing models.

References	Dataset	Parameters	Classes	Models/Methods	Accuracy (%)	AUC (%)	Precision (%)	F1-Score (%)
Pang et al. [37]	Shandong Provincial Hospital	–	3	DenseNet + AdaBoost	89.85	–	–	–
Naseer et al. [27]	LUNA-16	–	2	U-Net + AlexNet-SVM	97.98	–	97.53	97.70
Han et al. [3]	Peking University Cancer Hospital	144	2	VGG16	84.10	90.30	74.40	–
Wang et al. [6]	SUCC	–	4	Weakly supervised DL + RF	97.30	85.60	–	–
Demiroğlu et al. [45]	Chest CT-Scan images	20	4	DarkNet53 + DenseNet201	98.86	98.70	–	–
Nahiduzzaman et al. [46]	Chest CT-Scan images	0.53	2	LPDCNN-Ridge-ELM	99.70 ± 0.45	–	–	–
Nahiduzzaman et al. [46]	Chest CT-Scan images	0.53	4	LPDCNN-Ridge-ELM	98.40 ± 0.82	–	–	–
Proposed Method	Chest CT-Scan images	304	2	CNN + SVD + Ensemble Learning	100.00	–	100.00	–
Proposed Method	Chest CT-Scan images	304	4	CNN + SVD + Ensemble Learning	99.49	–	100.00	–

In the realm of four-class classifications (ADC, LCC, normal, and SCC), Demiroglu *et al*. and Nahiduzzaman *et al*. conducted notable studies, achieving accuracies of 98.86% and 99%, respectively. Nevertheless, the proposed model outperformed the other methods, with an accuracy of 99.49%. Notably, the system achieved 100% accuracy in classifying normal cases across all classifiers, a result validated by Grad-CAM. Furthermore, in comparison to existing SOTA techniques, the suggested model provided improved resilience and dependability due to its thorough 5-fold cross-validation.

### 4.7. Discussion

This study introduces a novel hybrid ensemble method for lung cancer classification, combining Singular Value Decomposition (SVD) with Convolutional Neural Networks (CNN) to effectively extract and classify key features from CT scan images. The approach begins with an image preprocessing phase, where Contrast Limited Adaptive Histogram Equalization (CLAHE) is applied to enhance the image quality. This crucial step ensures that the input data is of high quality, setting a solid foundation for the subsequent analysis. Following image enhancement, a customized CNN-SVD architecture is used for feature extraction and selection. This hybrid model is designed to capture complex patterns in the lung cancer (LC) CT scan images. The CNN efficiently extracts high-level spatial features, while the SVD algorithm performs dimensionality reduction by selecting the most informative and uncorrelated features. This combination addresses common challenges in medical imaging, such as redundancy and feature correlation, computational efficiency ultimately improving classification performance. From the extracted features, 100 prominent ones are selected using SVD to refine their quality and ensure they are compatible with the downstream machine learning algorithms. To classify the lung cancer cases, the model employs a set of well-established machine learning algorithms: Gaussian Naive Bayes (GNB), Gradient Boosting Machine (GBM), k-Nearest Neighbors (KNN), Support Vector Machine (SVM), and Random Forest (RF). These classifiers, combined in an ensemble learning framework, contribute to the robustness of the model by generating a collective prediction from multiple models. This ensemble approach not only reduces the risk of overfitting but also improves the model’s generalizability to new data. The ensemble learning framework is further enhanced by the inclusion of 5-fold cross-validation, which demonstrates the model’s reliability and stability. This cross-validation ensures that the model’s performance is consistent across different subsets of the data, thereby increasing its robustness. Additionally, Grad-CAM is employed to visualize and interpret the decision-making process of the CNN. The high classification accuracy, coupled with visual interpretability through Grad-CAM, provides a strong foundation for real-world clinical applications, potentially reducing the risk of misdiagnosis and enhancing clinical decision-making.

However, some assumptions were made during the development of the model. It was assumed that the dataset used is representative of the broader population of lung cancer patients, capturing a wide range of tumor types and imaging conditions. It was also presumed that data heterogeneity introduced by different image capturing techniques would not significantly affect the model’s performance. The CNN and SVD combination was expected to retain the most critical features for classification, while reducing dimensionality and maintaining high accuracy in both binary and multiclass classification tasks.

Despite these assumptions, potential threats remain. For instance, data source bias could impact the model’s generalizability, as images from limited sources may not represent the broader population of lung cancer patients. Moreover, the model was developed specifically for CT scan images, and its performance on other imaging modalities, such as MRI or X-ray, remains uncertain. Additionally, factors such as patient demographics (age, sex, ethnicity, and socioeconomic status) and co-morbidities (e.g., chronic obstructive pulmonary disease, diabetes, or hypertension) were not explicitly accounted for in the model. These external factors could influence the model’s performance in real-world clinical settings.

#### 4.7.1. Limitations and future recommendations.

Although this study achieves significant results outscoring all the state-of-the-art results, it has few limitations. For example, the model’s performance may be affected when applied to unseen datasets or different clinical settings, as the dataset used in this study was sourced from a single publicly available repository with limited diversity in imaging conditions, scanner types, and patient demographics. Furthermore, owing to limited computational resources, pretraining on large-scale datasets such as ImageNet could not be conducted. Prior research has shown that fine-tuning pre-trained models can accelerate the training process and enhance performance. For future improvements, the use of additional advanced pre-processing techniques (e.g., Bengraham) to further enhance input data quality is recommended. Additionally, integrating transfer learning from large-scale datasets with the current network architecture should be investigated to leverage the strengths of both approaches. Expanding the model’s evaluation on diverse datasets and clinical environments will also be crucial to validate its generalizability for lung cancer classification using CT images.

## 5. Conclusion

The experimental results for multiclass classification using the hybrid CNN-SVD-based ensemble model demonstrate remarkable performance metrics, including an accuracy rate of 99.49%, an AUC of 99.73%, a precision of 100%, a recall of 99%, and an F1 score of 99%. Similarly, for binary class classification, all the performance parameters achieved a perfect score of 100%. These outcomes underscored the exceptional performance and robustness of our proposed approach. These successful tests demonstrated the practical utility and reliability of the model beyond controlled experimental conditions. In conclusion, the hybrid CNN-SVD-based ensemble model, augmented with Grad-CAM, not only achieved outstanding results in both binary and multiclass lung cancer classification but also offered enhanced interpretability, making it a strong candidate for clinical application in medical image analysis. This research holds significant promise for advancing lung cancer diagnosis and treatment planning.

**Summary Points:**

- This study introduces a novel lung cancer (LC) detection method tailored for binary and multiclass classification using publicly available CT scan images of patients with lung cancer.
- CLAHE was employed to enhance distinctive features, and CNN-SVD was used to facilitate feature extraction and dimensionality reduction, optimizing feature representation.
- LC classification achieves remarkable metrics with an accuracy, AUC, precision, recall and F1 score of 99.49%, 99.73%, 87.5%, 100%, and 99%, respectively.
- In binary class (cancer and non-cancer) classification, all performance indicators attained a perfect score of 100%.
- Gradient-weighted Class Activation Mapping (Grad-CAM) was integrated to provide interpretability by visualizing critical regions in CT scans that influence predictions, enhancing model robustness and transparency.
